# Extended Roles in Healthcare Delivery: What Is the Role of the Laboratory in Addressing Ethnicity-Related Healthcare Disparities?

**DOI:** 10.3390/diagnostics15222919

**Published:** 2025-11-19

**Authors:** Aman Kaur More, Nathan Lorde, Tejas Kalaria, Rousseau Gama

**Affiliations:** 1 Blood Sciences, Black Country Pathology Services, The Royal Wolverhampton NHS Trust, Wolverhampton WV10 0QP, UK; tejaskumar.kalaria@nhs.net (T.K.); rousseau.gama@nhs.net (R.G.); 2 Clinical Biochemistry, University Hospitals Birmingham NHS Foundation Trust, Birmingham B15 2GW, UK; nathan.lorde@nhs.net; 3 School of Medicine and Clinical Practice, University of Wolverhampton, Wolverhampton WV1 1LY, UK

## 1. Introduction

The drivers of healthcare inequalities, often interlinked, include socioeconomic status, geographic barriers, gender, age, and ethnicity [1]. These factors limit access to healthcare and bias medical care, contributing to delayed diagnosis and poorer outcomes [2,3,4,5]. Such inequalities are well-recognised; in England, for example, the National Health Service (NHS) launched its Healthcare Inequalities Improvement Programme in 2021 to address disparities in healthcare access, experience, and outcomes across the population [6].

An estimated 70–80% of patient diagnoses and 95% of all clinical pathways rely on pathology results [7]. Clinical laboratories may therefore play an important, yet underexplored, role in mitigating these disparities. The recent removal of the ethnicity correction from estimated glomerular filtration rate equations [8,9] has underscored the need to identify and eliminate ethnic disparities in laboratory healthcare delivery. This editorial focuses on the role of clinical laboratories in addressing ethnicity-related healthcare disparities within cosmopolitan populations. Ethnicity, race, and ancestry are often used interchangeably but represent distinct, though sometimes overlapping concepts. Ethnicity refers to groups sharing a common cultural and social identity; race is a social construct used to group people, largely but not exclusively, on physical characteristics; and ancestry denotes genetic lineage and geographical origins [10,11]. Ethnicity is routinely collected in NHS electronic health records (EHR), though not in all cosmopolitan healthcare systems, and may be used to identify disparities in access, outcomes, and service use. Broad ethnicity or race categories are often used as convenient proxies for underlying genetic diversity and environmental factors affecting laboratory results. For example, a gene affecting neutrophil levels is more prevalent in individuals of African descent, contributing to benign neutropenia [12]. However, ethnicity is not a perfect proxy for genetic diversity. Substantial genetic heterogeneity exists within ethnic groups, and descriptors like race, ethnicity, or geographic origin do not capture the continuum of individual variation. In the USA, The National Academies of Sciences, Engineering, and Medicine therefore recommended shifting away from using race, ethnicity, and geographic origin as proxies for genetic ancestry groups [13]. Although not ideal, until genomic databases include broader population coverage, ethnicity remains the most relevant construct for current routine laboratory practice.

This editorial focuses on reducing ethnicity-related healthcare inequalities. This includes addressing potential ethnicity bias in test development and interpretation, such as removing inappropriate ethnicity adjustments [8,14], and improving laboratory access for populations that are traditionally difficult to engage [15,16], with the aim of delivering equitable healthcare.

## 2. Inclusive Reference Intervals for Diverse Populations: Ethnicity-Specific or Personalised?

Reference intervals (RIs) for laboratory tests are typically defined as the central 95% ranges in a healthy (reference) population. Traditionally, laboratories apply “one-size-fits-all” or universal Ris, with adjustments for factors like sex and age. Laboratory results from minority ethnic groups are typically interpreted using RIs derived from majority ethnic populations. Yet ethnicity-related biological differences in biomarkers exist (Table 1) [8,12,17,18,19,20,21,22,23,24,25,26,27,28,29,30,31,32]. When applied to minority ethnic groups, generic RIs may misclassify results from healthy individuals as abnormal (outside RI), resulting in unnecessary further investigation and patient anxiety, or classifiy results from individuals with disease as normal (within RI), delaying diagnosis and treatment. Ethnicity-specific RIs may therefore reduce ethnicity-related misclassification bias and improve equity. However, the clinical significance of ethnic differences in biomarkers remains incompletely established. The impact of ethnicity-related differences, for example, in HbA1c irrespective of glycaemia [33], lipid profiles [34], cardiac enzymes and serum magnesium [35,36], on clinical management remains to be further studied and clarified.

Although ethnicity is a useful indicator of disparities, ethnicity-specific RIs must be applied with caution. They risk overlooking variation within ethnic groups and may inadvertently reinforce racial bias. Additionally, not all laboratories have sufficient data to derive robust ethnicity-specific RIs, particularly for minority and paediatric subpopulations.

A promising alternative is personalised RIs—precision ranges tailored to an individual’s characteristics and incorporating multiple covariates, including age, sex, comorbidities, pregnancy status, ethnicity, and relevant genetic data. Individualised RIs derived from big datasets could reflect a more comprehensive picture of physiology and pathology, thereby reducing both false positives and false negatives across all populations [60,61]. Evidence of improved outcomes, however, remains limited [60]. Personalised RIs could be enabled by artificial intelligence (AI) using historical data stored in EHRs. Yet significant challenges remain. Large datasets required to derive personalised RIs are sparse, and personalised RIs are dependent on patients’ prior results [60]. Existing information technology infrastructure in most laboratories and hospitals is not equipped to implement such RIs [62]. AI may also perpetuate inequality [63] by reproducing biases from training data or design processes [64,65,66,67,68,69,70,71,72,73]. Avoiding bias in machine learning and AI is complex and currently without definitive solutions [74,75,76,77,78,79,80].

Personalised RIs are therefore unlikely to become a reality in the near future. Meanwhile, laboratories, especially those serving ethnically diverse populations, should remain alert to the limitations of traditional RIs and aware of ethnicity-related differences when assessing the need for ethnicity-specific intervals. Where indicated, this could include adopting suitable ethnicity-specific RIs from literature or deriving RIs for the local population using indirect data-mining approaches from laboratory and hospital electronic records [8].

## 3. Improving Access to Laboratory Healthcare Services

In addition to addressing ethnicity bias in test development and interpretation, all individuals should have equitable access to laboratory services. Ethnic minority populations are more likely to encounter barriers to accessing healthcare such as limited transportation, mistrust of healthcare systems, a lack of culturally appropriate services, and language differences [81,82,83]. Improving laboratory access for underserved groups through point-of-care testing (POCT) and extension of phlebotomy services offers potential mechanisms to reduce ethnic disparities in laboratory healthcare delivery.

POCT involves performing tests near the patient in a variety of clinical settings using standalone devices, mobile laboratories, or home-testing approaches. POCT has traditionally been used to provide test results in real time so that patients may quickly be triaged or given appropriate treatment, yielding improved clinical and economic outcomes compared to laboratory testing [84,85]. POCT, however, has also been used to reach individuals who may not engage conventional healthcare settings, including in places of worship, community centres, community pharmacies and primary care practices, for both diagnosis and optimisation of care [16,72,86,87,88].

The convenience and improved accessibility of POCT must be balanced with assurances of analytical quality and operator competence, within a framework of robust clinical governance that meets quality standards [89]. Clinical laboratories are uniquely positioned to ensure that POCT is safe, accurate, precise, and clinically meaningful.

Outreach phlebotomy directly addresses issues of transport, mobility, and geographical isolation that may disproportionately affect individuals from ethnic minorities. It would enable early detection and management of diseases, ultimately reducing ethnic laboratory health disparities, although its effectiveness and cost-effectiveness have not been systematically studied. Home-based sampling offers a discreet and accessible alternative that may overcome modesty-related barriers that deter individuals in certain minority populations from seeking healthcare services [90,91].

Strategies to achieve equitable access to laboratory healthcare services are more likely to succeed when developed in partnership with community and religious organisations, which can help bridge cultural barriers. For example, a successful outreach programme for hepatitis C testing among Pakistani immigrants in Scotland used mosques and women’s centres for education and testing [72]. Similarly, association with religious organisations improved access to and uptake rates of COVID-19 vaccinations in minority communities [92]. Linguistic barriers also reduce participation in preventative health programmes [93]; providing multilingual education and translated resources may enhance engagement and counter misinformation known to deter healthcare utilisation [72,94,95].

## 4. Conclusions

Ethnicity-related disparities in laboratory healthcare provision arise from multiple sources, including bias in test development and interpretation and reduced engagement with healthcare services due to mistrust and cultural barriers. In the short term, reducing these inequalities requires removal of inappropriate ethnicity-based equations and adoption of clinically relevant ethnicity-specific RIs. In the medium term, engaging ethnic minority community leaders and organisations in the development of health services, including outreach phlebotomy and laboratory testing, can help ensure that services are accessible, culturally appropriate, and continually evaluated for their impact on disparities. In the long term, awareness that AI models used in diagnostics may amplify existing biases must guide the development and application of AI methods that actively mitigate inequity. Laboratory medicine professionals, in collaboration with clinical and community stakeholders, are ideally positioned to implement several key strategies that address ethnicity-related disparities in laboratory healthcare delivery.

## Figures and Tables

**Table 1:** Examples of ethnicity-related differences in biomarkers.

Laboratory Test	Ethnicity-Related Differences
Haemoglobin	Adults from Black ethnicities have lower haemoglobin and MCV compared to age- and sex-matched White individuals. Higher prevalence of certain benign genetic conditions, for example, α-thalassemia trait, are believed to account for approximately a third of these differences, whereas iron status and other environmental factors may contribute towards the remaining [17,20,37].
White blood cell (WBC) count	African Americans and non-Hispanic Asians have lower WBC count than individuals of European and Hispanic ancestries [8].
Neutrophil count	Individuals of African and Arab ethnicities have lower neutrophil counts. This has often been described as benign ethnic neutropenia when an ethnicity non-specific RI is used. It is not associated with an increased risk of infection. A common Duffy antigen (rs2814778-C variant in the promoter of ACKR1 gene, Duffy-null), prevalent in individuals of African ancestry, is believed to be a large contributor (allele frequency of 0.96 in individuals from sub-Saharan Africa compared to 0.0006 in individuals of European ancestry). The loss of ACKR1 on the cell surface leads to neutrophil sequestration in tissues, lowering circulating neutrophils. However, in Arabs, the mechanism is less clear [12,20,21,38].
Ferritin	Men and women of African ancestries have higher serum ferritin compared to those of White ancestries; however, the exact mechanism is unclear. Contributors include higher iron stores because of dietary factors, genetic differences in iron handling, and subclinical inflammation-related ferritin elevation [37].
Transferrin Saturation	Men and women of African ancestries have lower serum transferrin saturation compared to those of White ancestries. Despite having higher serum ferritin, a larger proportion of individuals from African ancestries would be classified as having iron-deficiency based on transferrin saturation cut-offs. The difference narrows down but persists despite excluding individuals with possible iron deficiency or iron-deficient erythropoiesis of inflammation or chronic diseases indicating contribution from genetic factors like α -thalassaemia [37].
Creatinine and eGFR	Black individuals have higher serum creatinine, whereas certain Asian groups have lower serum creatinine compared to White ethnicities. These are primarily due to differences in muscle mass, but also dietary meat intake and tubular secretion of creatinine. However, ethnicity adjustment in estimated glomerular filtration rate (eGFR) equations may potentially overestimate GFR in Black individuals; thus, newer equations estimate eGFR without including ethnicity [8,14,19].
Total protein and globulin	Individuals of Asian ethnicities have higher total protein, globulin, and protein subfractions compared to those of White ethnicities [19]. These may be related to differences in immunoglobulins and binding proteins due to chronic antigenic exposure patterns and genetic factors [19,22].
Albumin	Black men and women have lower albumin compared to their White counterparts. The reasons are not fully understood but may include differences in albumin metabolism because of gene polymorphisms and subclinical effects of differences in nutrition and inflammation [17].
High-density lipoprotein (HDL) cholesterol	South Asians tend to have lower HDL cholesterol, whereas individuals of African ancestry generally have higher HDL cholesterol compared with White individuals. These differences arise from a combination of genetic factors (differences in cholesteryl ester transfer protein (CETP) activity), as well as obesity, insulin resistance, diet, alcohol intake, and physical activity [39,40,41].
Triglycerides	The ethnicity pattern of fasting triglycerides is the inverse of the HDL pattern: individuals of African ancestries have lower levels compared to those of White individuals, whilst South Asians have higher levels. Ethnic differences in central adiposity and insulin sensitivity are primary determinants of the differences with contribution from genetic polymorphisms in APOA5 and APOC3 [39,40,41].
Lipoprotein (a)	African ancestry individuals have the highest Lipoprotein (a) (Lp(a)) level (2–4-fold higher than Whites) followed by South Asian, White, Hispanics, and East Asian individuals. A larger proportion of Black and South Asian individuals exceed ethnicity non-specific risk thresholds for Lp(a). Individuals of African ancestries have a higher frequency of small-apo(a) isoform alleles, whereas East Asians have large isoform alleles. Therefore, African ancestry individuals produce more Lp(a) particles compared to East Asians. Additionally, the heritability of Lp(a) varies depending on ethnicity [42,43].
C-reactive protein (CRP)	Healthy Black men and women have higher CRP than their non-Hispanic White and Mexican American counterparts. Asian women have lower CRP than White women. The differences in socio-environmental factors, especially diet and central obesity, and CRP gene polymorphisms are possible contributors [17,23].
Alanine aminotransferase (ALT)	ALT levels in Mexican Americans are higher than those in White ethnicities, who in turn have higher ALT levels than Black ethnicities. The differences in AST are similar but less pronounced. Metabolic dysfunction-associated steatotic liver disease (MASLD) and genetic factors contribute to this. Hispanic populations have a higher frequency of PNPLA3 risk alleles associated with hepatic adiposity whereas Black ethnicities have low frequency of risk alleles and higher frequency of protective alleles [19,24,25]. Ethnicity differences have also been demonstrated in children; specifically, East Asian children have lower ALT [26].
Creatine kinase (CK)	CK levels are substantially higher in individuals of Black ethnicities compared to those of White and Asian ethnicities. This is believed to result from higher muscle mass in individuals of Black ethnicities and genetic variants affecting the CK set point [27,28].
Magnesium	Lower magnesium levels are seen in Black and South Asian individuals [35,36], a fact which also extends to South Asian children [26,44].
Amylase	“Ethnic hyperamylasaemia” is well recognised and benign—individuals of Black and Asian ethnicities have higher salivary and pancreatic amylase levels compared to those of White ethnicities. Genetic differences in AMY1 (salivary amylase) gene copy number are thought to be the main cause of this. However, those of ancestries with historically high starch diets have more AMY1 copy numbers. This difference is also seen in children [31,45].
Glycated haemoglobin (HbA1c)	HbA1c levels are higher in non-diabetic Black, South Asian, and Hispanic individuals compared to those in White individuals [46]. Black children with type 1 diabetes have been reported to have higher HbA1c than their White counterparts, and this is independent of mean blood glucose [47,48]. Postulated mechanisms are the effects of haemoglobin variants on HbA1c measurement, shortened erythrocyte lifespan, and genetic factors affecting glycation rates—for example fructosamine-3-kinase activity.
25-hydroxyvitamin D and parathyroid hormone (PTH)	Individuals of African and South Asian ancestries have significantly lower 25-hydroxyvitamin D concentrations than their White counterparts. South Asians living in northern latitudes often have very low vitamin D levels. PTH is higher in individuals of Black and South Asian ethnicities [49]. The differences are believed to result from interaction of genetic, sociocultural, and environmental factors. Higher melanin in darker skin decreases ultraviolet type B (UVB) mediated vitamin D synthesis. Sociocultural practices of full-length clothing, limited sun exposure, and limited intake of D-fortified foods or supplements may also be contributing factors [50,51]. Also, a common genetic variant in vitamin D–binding protein well recognised in Black Americans leads to lower measured total 25-hydroxyvitamin D, though the level of bioavailable vitamin D is the same as in their White counterparts [52]. This might explain the lower incidence of osteopenia and fractures in Black Americans compared to their White counterparts despite lower 25-hydroxyvitamin D (“Vitamin D paradox”) [49].
Thyroid-stimulating hormone (TSH)	Inland Asian populations have higher TSH, whereas African ancestry populations have slightly lower TSH compared to White ethnicities. There could be multiple explanations for these differences, including allele frequency differences in dual oxidase 2 (DUOX2) or deiodinase DIO1/DIO2 genes, lower thyroid autoimmunity in African ancestry populations, and lower iodine availability in inland Asian populations [53,54].
N-terminal pro-B-type natriuretic peptide (NT-proBNP)	NT-proBNP levels are approximately 30 to 50% lower in African Americans, lower in Hispanics, and higher in American Indian and East Asian populations compared to their age-matched White American counterparts. Polymorphisms affect genes coding atrial natriuretic peptide (ANP) and B-type natriuretic peptide (BNP) (NPPA/NPPB genes), neprilysin or natriuretic peptide clearance receptor. Higher prevalence of obesity in Black and Hispanic populations results in higher clearance of natriuretic peptides by adipose tissue (the so-called “natriuretic handicap”—higher rates of hypertension and heart failure but with low circulating natriuretic peptides as described in the literature). Hence, the use the non-specific cut-offs that do not account for ethnicity could result in underdiagnosing heart failure in Black individuals [55,56,57].
Prostate-specific antigen (PSA)	Black men without prostate cancer have higher baseline PSA levels compared to White and Hispanic men, whereas Asian men have lower levels. Racial differences in prostate size do not account for these differences. Higher testosterone levels and androgenic activity (shorter CAG trinucleotide repeats in the androgen receptor gene and a possibly less active variant of the CYP3A4 gene decreasing oxidative deactivation of testosterone) are postulated as contributors to this difference [32,58,59].

## Abbreviations

The following abbreviations are used in this manuscript:

NHS	National Health Service
EHR	Electronic Health Record
RI	Reference Interval
WBC	White Blood Cell Count
ACKR1	Atypical chemokine receptor-1
MCV	Mean corpuscular volume
eGFR	Estimated glomerular filtration rate
HDL	High-density lipoprotein
CETP	Cholesteryl Ester Transfer Protein
Lp(a)	Lipoprotein (a)
CRP	C-reactive Protein
MASLD	Metabolic dysfunction associated steatotic liver disease
PNPLA3	Patatin-like phospholipase domain-containing 3
CK	Creatine Kinase
AMY1	Alpha-amylase 1
HbA1c	Haemoglobin A1c
PTH	Parathyroid hormone
UVB	Ultra-violet B
TSH	Thyroid-stimulating hormone
DIO1/2	Iodothyronine deiodinase 1
ANP	Atrial natriuretic peptide
BNP	B-type natriuretic peptide
PSA	Prostate Specific Antigen
CYP3A4	Cytochrome P450 3A4
POCT	Point-of-care testing
AI	Artificial Intelligence
